# Robust Line Feature Matching via Point–Line Invariants and Geometric Constraints

**DOI:** 10.3390/s25102980

**Published:** 2025-05-08

**Authors:** Chenyang Zhang, Yunfei Xiang, Qiyuan Wang, Shuo Gu, Jianghua Deng, Rongchun Zhang

**Affiliations:** 1 School of Civil Engineering and Architecture, Changzhou Institute of Technology, Changzhou 213032, China; zhangchenyang@czu.cn (C.Z.); dengjh@czust.edu.cn (J.D.); 2 School of Computer Science and Engineering, Nanjing University of Science and Technology, Nanjing 210014, China; 3 Department of Geomatics Engineering, Nanjing Forestry University, Nanjing 210037, China; 4 College of Artificial Intelligence and Automation, Hohai University, Changzhou 213200, China; wangqy_16@163.com; 5 School of Geology and Geomatics, Tianjin Chengjian University, Tianjin 300384, China; rczhang@tcu.edu.cn

**Keywords:** line feature matching, point–line invariant, matching matrix, geometric constraint

## Abstract

Line feature matching is a crucial aspect of computer vision and image processing tasks, attracting significant research attention. Most line matching algorithms predominantly rely on local feature descriptors or deep learning modules, which often suffer from low robustness and poor generalization. In response, this paper presents a novel line feature matching approach grounded in point–line invariants through spatial invariant relationships. By leveraging a robust point feature matching algorithm, an initial set of point feature matches is acquired. Subsequently, the line feature supporting area is partitioned, and a constant ratio invariant is formulated based on the distances from point to line features within corresponding neighborhood domains. Additionally, a direction vector invariant is also introduced, jointly constructing a dual invariant for line matching. An initial matching matrix and line feature match pairs are derived using this dual invariant. Subsequent geometric constraints within line feature matches eliminate residual outliers. Comprehensive evaluations under diverse imaging conditions, along with comparisons to several state-of-the-art algorithms, demonstrate that our proposal achieved remarkable performance in terms of both accuracy and robustness. Our implementation code will be publicly released upon the acceptance of this paper.

## 1. Introduction

In the rapidly evolving field of computer vision and image processing, line features consistently play a pivotal and indispensable role [1]. As fundamental visual elements, they serve as the “skeletal structure” of images, encoding critical geometric and semantic information that underpins higher-level visual understanding [2,3]. Moreover, in contrast to pixel-level representations, line features provide a compact yet highly discriminative encoding of visual content, making them a powerful means of translating complex real-world scenes into a form that is both manageable and semantically meaningful.

As previously discussed in the context of image processing and computer vision, line features constitute the foundational framework of these fields. They act as essential structural frameworks that enable machines to parse visual complexity through hierarchical abstraction, effectively capturing the structural organization of objects and scenes. This characteristic remains consistent across various applications, whether identifying building edges in aerial imagery, detecting contours of mechanical parts in industrial inspection, or recognizing object boundary information in human-engineered environments [4,5]. The extraction and utilization of line features significantly enhance the efficiency and accuracy of numerous computational tasks, including, but not limited to, object detection and tracking [6,7] with higher precision than region-based methods in occlusion scenarios, reducing drift error through line-enhanced bundle adjustment of simultaneous localization and mapping (SLAM) [8,9], image segmentation, and scene understanding, thereby improving boundary accuracy compared to pure pixel classification approaches [10,11], and maintaining robustness in the terms of pose estimation [12]. These improvements stem from the inherent property of line features to encapsulate essential semantic information, which proves more robust and discriminative compared to individual pixel values or other elementary visual features (e.g., point).

Current line features description and matching can be broadly categorized into two main paradigms: traditional statistical local feature descriptors matching methods and deep learning-based matching models. These statistical-based descriptors approaches typically characterize line segments by aggregating and analyzing the pixel gradient or intensities within the line feature supporting region [2]. Most statistical-based line descriptors were partially inspired by the descriptor present in [13], in which the authors exploited image gradients in local subregions to formulate line feature descriptors according to the model of SIFT feature descriptor [14]. They also presented the line descriptor by exploiting the mean values and standard deviations from image gradients for line feature description. While most matching algorithms demonstrate considerable robustness against various image pairs transformation cases, they exhibit limitations in handling rotational variations and diverse matching scenarios.

On the other hand, deep learning-based models offer the advantage of combining both local and global contextual information for semantic line segment description. Learning-based line segment description approaches lies in learning the line segment representations locally or globally based on designed networks with loss functions and prepared training datasets. However, learning-based approaches present two significant challenges: (i) Their performance is heavily dependent on the quality and diversity of the training sample dataset. It is not easy to know whether they will perform well on other evaluation data since most of these networks are trained using their prepared datasets. (ii) They require substantial computational resources, typically necessitating GPU acceleration for practical implementation. Some practical applications often rely on limited computing power, which greatly limits the application of learning-based line matching algorithms.

To avoid the robustness issues of line feature matching algorithms based on feature descriptor and the generalization problems of line matching algorithms based on deep learning models, we have developed a new point–line feature invariants to perform line feature matching in this paper. Our approach offers two key advantages. Firstly, it significantly improves the robustness of image matching under challenging conditions such as rotation, scale variation, illumination changes, and viewpoint transformations, etc. Secondly, in contrast to other feature matching algorithms that are based on deep learning models or feature descriptors, our method operates without the need for GPU acceleration or feature descriptors. The main contributions of this work are summarized as follows:

- The proposed method leverages a fundamental geometric property to construct a new invariant of point–line features for line feature matching. We divide the line feature support area into left and right support regions, and construct invariants based on the constant distance ratio between point features in the support area and corresponding line features, thus completing line feature matching on any image pair.
- To enhance the precision and reliability of our line feature matching, we incorporate the directional vector of line features within the 2D image coordinate system as an additional invariant for line feature matching. Then, by combining the distance ratio and weighting two types of matching invariants, we score any line feature matching pair and construct a two-dimensional score matrix to achieve initial line feature matching.
- Two geometric constraints between line features are introduced to refine the initial matching results. Specifically, the final line feature correspondences are obtained by filtering the initial matches based on the midpoint distance and endpoint distance between line pairs. Furthermore, comprehensive comparative studies and systematic ablation experiments are conducted to evaluate the performance of the proposed algorithm. We will release our code to facilitate the implementation of our algorithms for researchers in this community.

The structure of this paper is organized as follows: Section 2 provides a systematic review of contemporary line feature matching algorithms, critically analyzing their methodological approaches and identifying specific limitations that motivate our work. Section 3 details our proposal algorithm, presenting its theoretical underpinnings, architectural design, and core technical innovations. In Section 4, we conducted extensive experimental evaluations using diverse datasets, providing both quantitative metrics and qualitative comparisons to demonstrate the superiority of our approach over existing methods. Section 5 provides an in-depth discussion of our algorithm’s current limitations and potential areas for improvement. Finally, Section 6 concludes the paper by summarizing our key contributions and outlining promising directions for future research on our work.

## 2. Related Works

Line feature matching presents significantly greater challenges compared to point feature matching [2,15], primarily due to several inherent difficulties: (1) The detection process often yields imperfect line segment representations, where physical lines may be fragmented into multiple discrete segments due to occlusion or noise. Even with advanced merging strategies, obtaining continuous and complete line representations remains challenging. (2) The inherent uncertainty in endpoint localization leads to complex many-to-many correspondence relationships between line segments across different images, substantially increasing the matching complexity.

Current line feature matching methodologies typically follow a four-stage pipeline: (1) initial line feature detection, (2) descriptor computation and representation, (3) feature correspondence establishment, and (4) geometric verification using constraints such as epipolar geometry or triangular constraints [16]. Based on their methodological approaches, existing line matching algorithms can be systematically categorized into three main paradigms: (i) local appearance-based methods, which rely on photometric information; (ii) geometric and topological-based approaches, utilizing structural relationships; and (iii) deep learning-based techniques, leveraging learned feature representations.

### 2.1. Line Matching Based on Local Appearance and Topological Information

Similar to the point feature matching strategy-based local descriptor, among the line features matching research process, an intuitive and fundamental idea is the design and construct line feature descriptors, which combine local appearances or relationships between line segments [17]. For example, Wang et al. [13] proposed the mean–standard deviation line descriptor (MSLD) which builds a description matrix based on the mean and standard deviation of intensity gradients in the neighborhood of the line. In many low or similar texture regions of images, MSLD possibly generates a less distinctive descriptor. Moreover, MSLD is sensitive to scale changes that are quite common in the feature matching area. MSLD may not perform perfectly in matching results for individual image situations, but the excellent matching results based on MSLD descriptors combining with topological information generate several related variants [18,19,20]. Regarding the problem of scale changes, Zhang et al. [18] improved the MSLD descriptor by combining local neighborhood gradients with global structural information of lines to enhance their distinctiveness. Zhang et al. [19] proposed the line segments matching algorithm of MSLD description based on the corresponding point constraint. Zhang et al. [20] designed and proposed the line strip descriptor (LBD), which calculated descriptors in the line support regions, and incorporates local appearance and geometric properties to improve line feature matching accuracy for low-texture scenes through pair-wise geometric consistency evaluation.

In addition to directly constructing feature local descriptors, some related researchers are also tempted to utilize geometric and topological information for line matching. Bay et al. [21] introduced a wide-baseline line matching method considering color profiles, topological structures, and epipolar geometry, and added more line feature matches iteratively by applying sidedness constraint. To enhance line matching results for wide baseline matching, B.Vogern et al. [22] proposed a strategy that introduces scale invariance into line descriptors by improving both Bay’s approach and the MSLD descriptor. By applying the topological information within line features, Li et al. [23] constructed the “Line Junction Line” structure, which connects intersection points of line features and intensity maximum points to form a local affine invariant region. Meanwhile, SIFT feature descriptors are then calculated within the local image region, replacing line feature descriptors for matching.

In some challenging scenarios, such as lighting changes and severe or sparse low-texture scenes, Lin et al. [24] designed the illumination–insensitive line binary (IILB) descriptor, which is based on band differences among multiple spatial granularities of line supporting region. The IILB descriptor needs lower computational costs, making it suitable for platforms with limited computational and storage resources. When facing low-texture stations for line matching, Wang et al. [25] proposed a semi-local feature (LS) approach based on the local clustering of line segments for matching wide-baseline image pairs. Unfortunately, this matching process is computationally expensive. To improve matching accuracy aiming at low-texture scenes or under uncontrolled lighting conditions, Lopez et al. [26] introduced a dual-view line matching algorithm that combines the geometric characteristics of lines, local appearance, and the structural context of line neighborhoods. It iteratively matches lines by utilizing structural information gathered from different line neighborhoods, ensuring a stable growth of the matched line segment set during each iteration. Wang et al. [27] proposed a unified approach that combines Canny edge detector and adopt the graph matching method using spectral technique for line detection and stereo matching, yielding promising results in un-textured scenes. To satisfy matching images pairs with large viewpoint changes, Wang et al. [28] presented a line segment matching method based on pair-wise geometric constraints and descriptors. They applied three pair-wise geometric constraints and one line pair-based Daisy descriptor similarity constraint to generate corresponding line pairs in the initial matching stage, with two pairs of corresponding lines contained in each corresponding line pair.

### 2.2. Line Matching Methods Jointing Point Feature

As point features matching have been extensively studied within the image processing and computer vision community, researchers have also attempted to use point features to construct point–line feature invariants to achieve line feature matching [29,30,31].

Schmid and Zisserman [29] proposed to first find point correspondences on the matched lines by the known epipolar geometry and then to average the cross-correlation scores over all the corresponding points as the line similarity for matching. The method needs to know the epipolar geometry in advance. Lourakis et al. [30] used two lines and two points to construct a projective invariant for matching planar surfaces with lines and points. But their method can hardly be extended to non-planar scenes and has high computational complexity. To reduce the time complexity of the line matching algorithm and improve its robustness, an affine invariant derived from one line and two points [15] and a projective invariant derived from one line and four points [31], which encode local geometric information between a line and its neighboring points. However, the point–line invariant proposed in references [15,31] highly depends on the accuracy and robustness of matched point features. To minimize the dependence of point and line feature invariants on the accuracy of point feature matching as much as possible, Jia et al. [32] developed a novel line matching method based on a newly developed projective invariant, named characteristic number (CN). They used the CN to construct original line correspondences and then applied a homography matrix to filter mismatched line features. According to our own practical experience, this algorithm performs very well in image scaling and rotation. In low-texture or weak-texture scenes, the number of matches may decrease due to the distribution and matching accuracy of feature point images.

In some cases, line matching can be complicated by occlusion, line breakage, which makes shape-based global descriptors less applicable, by using point features for auxiliary matching, relatively ideal results can sometimes be obtained. For example, in ref. [33], line feature matching is achieved by discretizing the line features and combining them with point constraints associated with the same name. In ref. [34], the SIFT is used to identify points with the same name, and the Freeman chain code algorithm is utilized to extract lines. The initial line matching is based on the positional relationships between dense matching points and lines in the search area. The matching results are then refined by optimizing the degree of coincidence between line segments, and the endpoints of lines with the same name are determined using kernel constraints.

### 2.3. Line Matching Based on Deep Learning Model

With the rapid development of deep learning, convolutional neural networks have become increasingly prominent in feature matching tasks due to their powerful ability to extract deep features or feature descriptor. The core idea of learning-based line segment description approaches lies in learning the line segment representations locally or globally based on designed networks with loss functions and prepared training datasets [2].

SOLD2 [35] was the first network to leverage deep learning for detecting and describing line features, while LineTR [36] constructs line feature word vectors using an attention mechanism, incorporating contextual information about the line features to achieve accurate line feature matching and visual localization. Song et al. [37] adopted a different approach by extracting discrete points along line segments, calculating the single-sided descriptors of these points based on the grid information, and applying a deep learning model or mechanism to aggregate these point descriptors into a single-sided abstract representation of the line. M. Lange et al. [38] utilized the Unreal Engine and multiple scenes provided for it to create training data. The training was performed using a triplet loss, where the loss of the network is calculated with triplets each consisting of three lines, including a matching pair and another non-matching line. Subsequently, M. Lange et al. [39] trained a neural network to compute a descriptor for a line, given the preprocessed information from the image area around the line. In the pre-processing step, they utilized wavelets to extract meaningful information from the image for the descriptor. The core idea of learning-based line segment description approaches lies in learning the line segment representations locally or globally based on designed networks with loss functions and prepared training datasets.

Therefore, designing the network and preparing the training and testing datasets are critical. Like deep learning-based line segment detection methods, the generalization ability of trained networks needs to be further validated, since most of them trained their networks using their prepared datasets.

## 3. Line–Points Projective Invariant

In this section, we will focus on introducing the construction detail of point–line feature projection invariants in this paper. We will respectively elaborate on the point–line invariants within the line feature support region (i.e., there are corresponding point features on both left and right sides of the line feature neighborhood), the invariants in the semi support region of the line feature (i.e., one side of the line feature neighborhood has corresponding point features, and the other side is the image edge or low-texture region), and the feature projection invariants of the line feature direction vector.

### 3.1. Line Feature Full-Neighborhood Invariant

**Figure 1:**
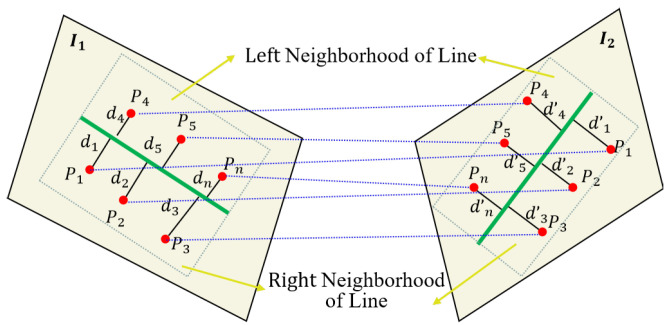
Schematic representation of point–line invariants in full-neighborhood line feature.

As shown in Figure 1, assume that features points $p_{1}^{I_{1}}$, $p_{2}^{I_{1}}$, $p_{3}^{I_{1}}$, ${\;p}_{n}^{I_{1}}$ and line feature *L*_1_ all lie on image *I*_1_, and these feature points are distributed on the left and right sides of the line feature *L*_1_. Such correspondence features points $p_{1}^{I_{2}}$, $p_{2}^{I_{2}}$, $p_{3}^{I_{2}}$, ${\;p}_{n}^{I_{2}}$ and line feature *L*_2_ all lie on image *I*_2_, and these feature points are distributed on the left and right sides of the line feature *L*_2_. Since $p_{i}^{I_{1}}$ and $p_{i}^{I_{2}}$ are coplanar in 3D space, the homogeneous coordinates of feature points are related by a homography matrix. Here, we considered a specific case in which the transformation between the neighborhood of line feature pairs satisfies the following relationship:

(1) $$p_{i}^{I_{1}}=sHp_{i}^{I_{2}}.$$

For any feature point belonging to the neighborhood of line features, the distance from the feature point to the line can be directly calculated by the coordinates of the point and the line feature equation, which can be expressed as $D_{left}^{I_{1}}$(*p*,*L*) or ${\;D}_{right}^{I_{1}}$(*p*,*L*) in Equation (2). Here, we clearly represent the distance of different points to lines within the neighborhood of line features on different images. As shown in Figure 1, note that the neighborhood of line feature we are talking about here refers to the rectangular region that we have formed with length of the line feature and half the length of the line feature, which is called the line feature neighborhood in this paper.

(2) $$D_{*}^{I}(P,L)=\tilde{L}$$

If the neighborhood of line feature is determined, we can determine whether the points belong to the neighborhood of the line feature by comparing the coordinates of the four vertices of the rectangle neighborhood and the coordinates of the point features. Assuming that we have calculated the distance from these points $\;(p_{i},p_{i}^{\prime })$ located in the neighborhood of the line feature to these lines (*l*_1_, *l*_2_) using Formula (2), and obtained a total of 4 sets of data result sequences on the left and right sides of each image line feature, respectively referred to as ***Q*_1_**, ***Q*_2_**, ***Q*_3_**, ***Q*_4_**.

(3) $$\left\{\begin{array}{c} Q_{1}:{\{\;D}_{1}^{left}(p_{1},l_{1}),D_{2}^{left}(p_{2},l_{1}),\ldots ,D_{n}^{left}(p_{n},l_{1})\}\;\;\;\;\\ \begin{array}{c} {Q_{2}:\{\;D}_{1}^{right}(p_{1},l_{1}),D_{2}^{right}(p_{2},l_{1}),\ldots ,D_{n}^{right}(p_{n},l_{1})\}\;\;\;\\ Q_{3}:{\{\;D}_{1}^{left}({p\prime }_{1},l_{2}),D_{2}^{left}({p\prime }_{2},l_{2}),\ldots ,D_{n}^{left}({p\prime }_{n},l_{2})\}\;\;\;\\ Q_{4}:{\{\;D}_{1}^{right}({p\prime }_{1},l_{2}),D_{2}^{right}({p\prime }_{2},l_{2}),\ldots ,D_{n}^{right}({p\prime }_{n},l_{2})\}\;\; \end{array} \end{array}\right.$$

Assuming there are matching points ($p_{i},p_{i}^{\prime }$) and corresponding line features ($l_{1}$,$l_{2}$) on two images, there is a mathematical relationship between the distance of point features in the neighborhood (i.e., left and right) of line features to the line:

(4) $$\left\{\begin{array}{c} \frac{D_{i}^{left}(p_{i},l_{1})}{D_{i}^{left}({p\prime }_{i},l_{2})}=\delta \\ \frac{D_{j}^{right}(p_{j},l_{1})}{D_{j}^{right}({p\prime }_{j},l_{2})}=\delta \end{array}\right.,$$

δ represents the ratio value, so we obtained the following point line feature invariant:

(5) $$\frac{D_{i}^{left}(p_{i},l_{1})}{D_{i}^{left}({p\prime }_{i},l_{2})}/\frac{D_{j}^{right}(p_{j},l_{1})}{D_{j}^{right}({p\prime }_{j},l_{2})}=1.$$

As a fact, due to the presence of image noise in the detection process of point or line features, as well as a certain number of incorrect matches in the matching process of point features. To reduce errors, we first continuously try to calculate the variance of line features, remove corresponding mismatches of point features, and then calculate the variance of each pair of data sequences (***Q****_i_*, *i* = 1, 2, 3, 4) until the variance meets a certain threshold. We retain the corresponding matching pairs of distance data sequences {***Q*′_1_**, ***Q*′_2_**, ***Q*′_3_**, ***Q*′_4_**}. To further reduce the error of point line features in image pairs, we construct point line invariants for any line feature within the entire neighborhood based on the average idea using the following Formulas (6) and (7):

(6) $$\frac{\sum D_{i}^{left}(p_{i},l_{1})/M}{\sum D_{i}^{left}({p\prime }_{i},l_{2})/M}/\frac{\sum D_{j}^{right}(p_{j},l_{1})/N}{\sum D_{j}^{right}({p\prime }_{j},l_{2})/N}=1,\;\;i\in \{{Q’}_{1},{Q’}_{2}\},\;j\in \{{Q’}_{3},{Q’}_{4}\}\;,$$

*M* and *N* represent the corresponding matching numbers of point features in the left and right neighborhood of line features, respectively. Division eliminates *M* and *N* to obtain Formula (7):

(7) $$\frac{\sum D_{i}^{left}(p_{i},l_{1})}{\sum D_{i}^{left}({p\prime }_{i},l_{2})}/\frac{\sum D_{j}^{right}(p_{j},l_{1})}{\sum D_{j}^{right}({p\prime }_{j},l_{2})}=1\;,\;\;i\in \{{Q’}_{1},{Q’}_{2}\},\;j\in \{{Q’}_{3},{Q’}_{4}\}\;.$$

### 3.2. Line Feature Semi-Neighborhood Invariant

**Figure 2:**
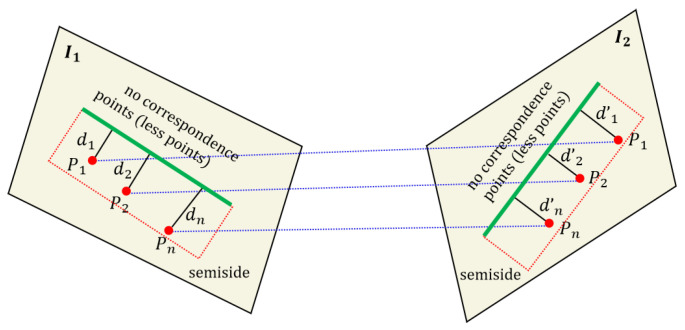
Visualization of point–line invariants for line feature matching in semi-neighborhood.

Due to the fact that some line features are located at the edges of the image, or the fact that on the other side of the image are areas with unclear grayscale changes such as the sky and white walls, this can result in half of the area not having corresponding feature points. As shown in Figure 2, assume that feature points $p_{1}$, $p_{2}$, $\;p_{n}$ and line feature *l*_1_ all lie on image *I*_1_, and these feature points are distributed on the semi-sides of the line feature. The corresponding feature points $p_{1}^{\prime }$, $p_{2}^{\prime }$, $p_{n}^{\prime }$ and line feature *l*_2_ all lie on image *I*_2_, and these feature points are distributed on semi-side of line feature *l*_2_. *d_i_*, *d*’*_I_* (*i* = 1, 2, … n) denote the distance from the corresponding point feature to the line. Here, no corresponding points denoting the number of point features in this area are very small or even 0.

In this case, we can only obtain point feature correspondences within half of the neighborhood, and we thus can only obtain sequences of two pairs of point features to the corresponding line features, which are still labeled as ***Q*_1_**, **Q_2_**. According to Formulas (1)~(5), we know that the ratio of the distance from each corresponding point to the line feature should be a constant value, represented by the formula:

(8) $$\frac{D_{1}^{semi}(p_{1},l_{1})}{D_{1}^{semi}(p_{1}^{\prime },l_{2})}=\lambda_{1}=\frac{D_{1}^{semi}(p_{1},l_{1})}{D_{1}^{semi}(p_{2}^{\prime },l_{2})}=\lambda_{2}\ldots \frac{D_{n}^{semi}(p_{n},l_{1})}{D_{n}^{semi}(p_{n}^{\prime },l_{2})}=\lambda_{n}.$$

For any line feature to be matched, we obtain a set of data sequences $q(\lambda_{1},\lambda_{2},\cdots ,\lambda_{n})$, using Formula (8). Due to incorrect matching of point features, these data sequences also have no obvious matching points, so there will be outliers in the data sequence q. Next, we only need to remove the outliers. We first find the median of the data sequence and then use three times the median as the interval to remove those abnormal proportion values. After which, we calculate the variance of the sequence and obtain the corresponding score as shown in Formula (9), which will be filled into our subsequent matching matrix. Note the variance of ***q*** here, and we will need to preset a threshold *k* to further exclude the ***q*** with outliers.

(9) $${socore}_{distance}=\left\{\begin{array}{c} 1-var(q),\;\;k=var(q),k\leq 0.7\\ \;\;\;0,\;k=var(q),k>0.7 \end{array}\right.$$

### 3.3. Line Feature Direction Vector and Matching Matrix

**Figure 3:**
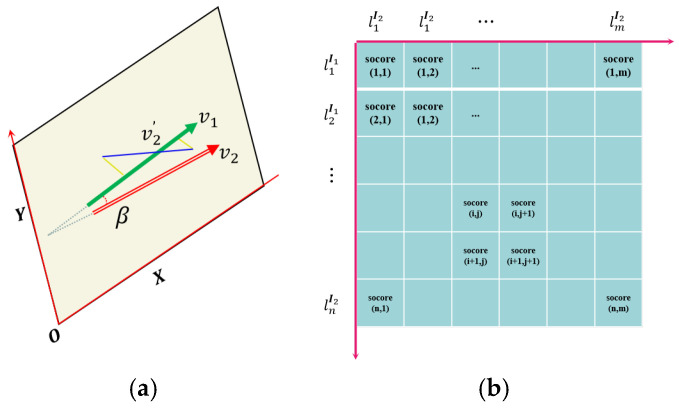
Schematic diagram of angel invariant for semi-neighborhood of line features and the scoring matrix. (**a**) The angel constraint between line features; (**b**) the scoring matrix.

As shown in Figure 3a, although the endpoint distance between the line feature matching pairs is relatively small, it is possible that the line matching pair is not the correct (e.g., ***v***_2_′). To further enhance the robustness and accuracy of line feature matching, we introduce the constraint of line segment direction vector here. As shown in Figure 3a, we establish an image plane coordinate system and calculate the line feature direction vector ***v_1_*** using the line endpoint coordinates. If the homography mapping is accurate and the definition and extraction of line features in the original image are precise, then theoretically, the matched lines should “coincide” at their corresponding positions in another image after homography mapping (here, coincidence refers to the correct matching of the projection positions of the same line in the space they represent in two images under an ideal projection relationship). At this point, the direction vector of line feature is denoted as ***v_2_***. The angle between the two is calculated using Formula (9):

(10) $$\alpha =<v_{1},v_{2}>=\frac{{v_{1}^{T}v}_{2}}{\left||v_{1}||\cdot ||v_{2}|\right|}.$$

We score based on the angle value of the linear direction vector, and our scoring rules are as follows:

(11) $${socore}_{angle}\left\{\begin{array}{c} 1,\alpha \;\epsilon \;[175,180]\;or\;[0,5]\;\\ \frac{\left|\alpha -90\right|}{90},\;\alpha \epsilon [5,175] \end{array}\right..$$

Based on the above constraints, we score each line feature pair and then construct a scoring matrix. The score matrix is shown in Figure 3b, where the rows and columns represent the line features contained in the image matching pair, and each row and column represents the score value corresponding to any two line features. The calculation of the score value here is based on Formulas (12) and (13):

(12) $${socore}_{ij}=w_{1}\;{*\;socore}_{angle}+w_{2}\;{*\;socore}_{distance},$$

(13) $$w_{1}+w_{2}=1.$$

Finally, we obtain the initial matching pairs of line features by traversing the score matrix.

### 3.4. Line Feature Filtering Based on Geometric Constraint

After filtering the matching matrix based on point–line feature invariants and corresponding line feature matching, an initial set of line feature correspondences is established. However, due to factors such as rotation, scaling, and inaccuracies in point feature matching between image pairs, some incorrect line matches may still exist. To address this, geometric properties of the line features are utilized to further refine and validate the matching results. The geometric constraints for matching line features still use the distance from the line endpoint to the corresponding matching line and the distance between the midpoints of two adjacent line segments to constrain them.

**Figure 4:**
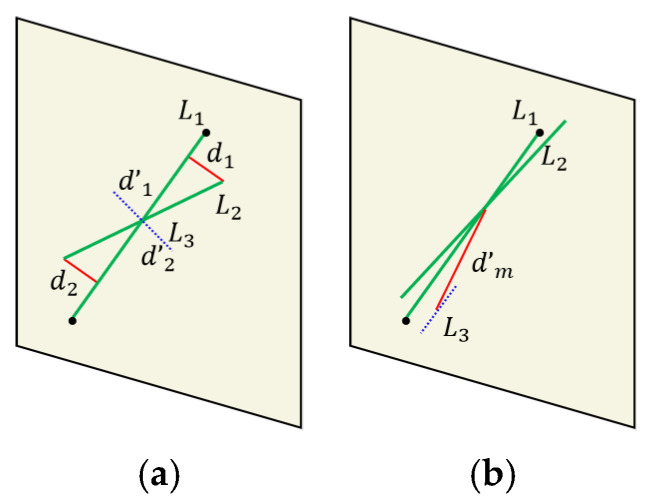
Diagrammatic representation of point–line invariant under geometric constraints. (**a**) endpoint distance constraint; (**b**) midpoint distance constraint.

As shown in Figure 4a, we construct the distance between the endpoints of the line feature and the line segment as a constraint and calculate it directly using Formula (14). Due to the constraint of angle invariants in Section 3.3, this can avoid the situation where some short line features (i.e., the blue dashed line in Figure 4a) are perpendicular to the line segment. In such cases, the distance between the endpoints is still relatively small, but the two are not line feature matching pairs.

(14) $$\left\{\begin{array}{c} d_{1}=\frac{\left|a*u_{1}+b*v_{1}+c\right|}{\sqrt{a^{2}+b^{2}}}\\ d_{2}=\frac{\left|a*u_{2}+b*v_{2}+c\right|}{\sqrt{a^{2}+b^{2}}} \end{array}\right.$$

**Figure 5:**
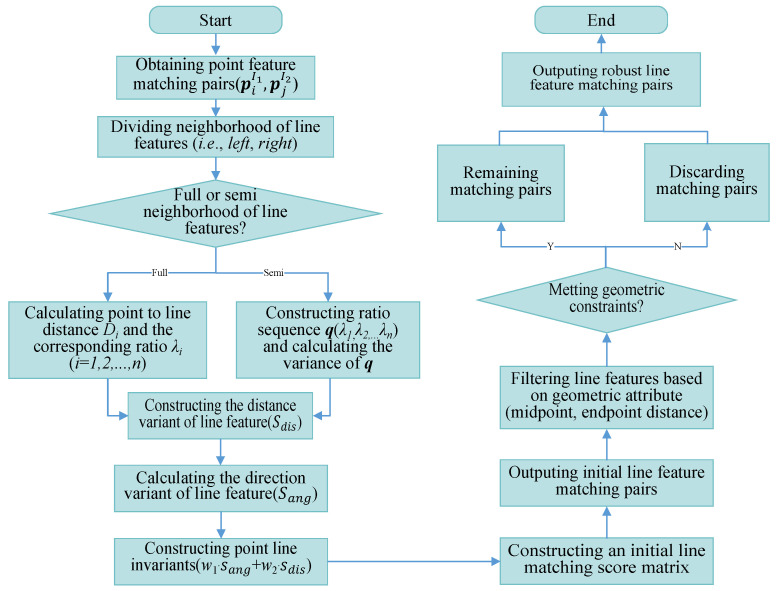
Schematic diagram of the proposed algorithm.

According to another situation shown in Figure 4b, there may be cases where both the endpoints and angles of the line segments are satisfied, but the midpoint distance of the line features is far away. Therefore, this type of line feature matching is actually an incorrect matching pair. In combination with this type of situation, we have added distance constraints between the midpoint of the line features (as shown by the red solid line in Figure 4b), which can eliminate the type of incorrect matching by adding distance constraints between the midpoint of the line features. Based on the detailed introduction of algorithm incorporating in this section, we have provided the complete algorithmic workflow in this paper, as shown in Figure 5.

## 4. Experimental Testing and Validation

**Figure 6:**
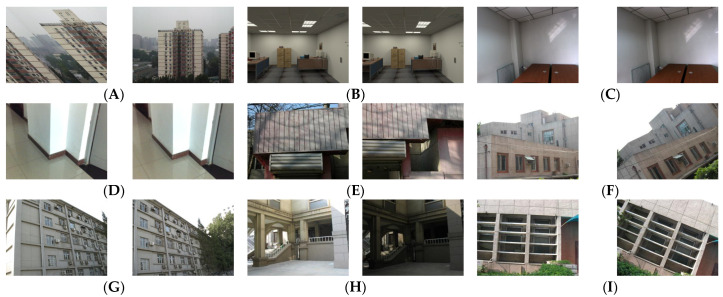
Validation of the proposed line feature matching algorithm across diverse scenarios [40,41]: (**A**) scale variation, (**B**) weak texture, (**C**,**D**) low texture, (**E**) occlusion, (**F**) rotation + low texture, (**G**) viewpoint change, (**H**) illumination variation, (**I**) rotation + weak texture.

To fully validate our proposal algorithm, we conducted a series of experiments and comparative studies in this section of our paper. We have selected some common line feature matching image pairs as the experimental data [40,41], which include low texture, scale changes, viewpoint changes, rotation, occlusion, and other situations. The above various image examples are shown in Figure 6A–I. It can be seen from the above examples of image pairs that these images contain almost all common encountered situations, and these examples are sufficient to verify the effectiveness and robustness of our algorithm. To weight the performance of the matching method quantificationally, we also define a variable *MP* as follows:

(15) MP=MC/MS

where *MS* is the total number of line feature matching pairs, *MC* is the number of correct matching pairs, and *MP* represents the matching accuracy. Our algorithm is implemented in programming of MATLAB R2018b. The operating system is an ordinary desktop machine that is equipped with a CPU without GPU acceleration, and 8 GB RAM.

### 4.1. The Parameter Settings for This Algorithm

**Figure 7:**
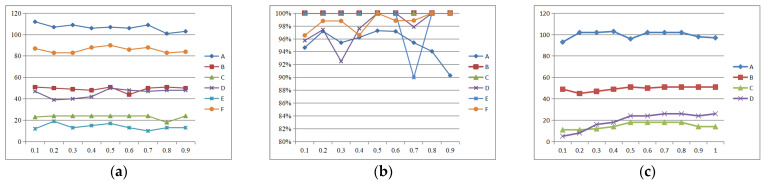
The number and accuracy of feature matching under different weight values and different thresholds. (**a**) Number of line feature matching pairs under different weights; (**b**) accuracy of feature matching under different weights; (**c**) the number of line feature matches under different thresholds.

To determine the values of *w*_1_ and *w*_2_ in Equations (12) and (13), we conducted validation experiments using data sequences (A)~(F) in the dataset. We varied the value of *w*_1_ back and forth between the range of 0.1 to 0.9 and obtained qualitative and quantitative results for Figure 7a and Figure 7b, respectively. Figure 7a represents the number of matching pairs of line features corresponding to each sequence under different weights, while Figure 7b represents the matching accuracy of line features corresponding to each sequence under different weights.

Based on the curve trends in Figure 7a,b, combined with the comparison of several data sequence results, when *w*_1_ and *w*_2_ are 0.5, the number and accuracy of feature matching basically reach the peak value. Therefore, we directly choose the weight value as *w*_1_ = 0.5, *w*_2_ = 0.5. We also selected several sets of image data sequences (A)~(D) to obtain the geometric threshold of point–line invariants in this paper, as shown in Figure 7c. It is not difficult to see that in order to obtain as many matching pairs as possible, we set the threshold k in this Equation (9) to 0.7.

### 4.2. Comparison Between Our Algorithm and Other SOTA Algorithms

We reproduced the line feature matching algorithm presented in ref. [23], named “Line Junction Line” invariant (LJL), which constructs a new line descriptor based on the SIFT features descriptor. Another state-of-the-art line matching algorithm, Hybrid Matching [27], uses Canny edge detector to detect lines and compute the corresponding LBD descriptor. Then, it can obtain robust and competitive matching performances under most scene data by graph matching. As a result, we selected the two matching algorithms for comparison and analysis in this study. To make a fair comparison, we use the line feature detection function interface in OpenCV. The experimental data used for this comparison includes image pairs (A)~(I), with the results summarized in Table 1. In Table 1, the bold values indicate the optimal result, while “—” denotes instances where the algorithm fails, and the matching experiment could not be completed.

According to the experimental results, we achieved optimal performance in 7 of 9 image sequences, as shown in Table 1. In contrast, the algorithm in ref. [27] fails for the image pairs (A), (G), and (I). The algorithm proposed in ref. [27] failed to complete experiments in individual image pairs, and their robustness performance is relatively weak. The method in ref. [23] completed all experiments, and their robustness performance is relatively weak in image pairs (B), (C), and (E). In the above image example scenario, our proposal was able to detect a rich number of feature points based on ORB feature points. Based on the point line feature matching invariant constructed in this paper, and taking into account the special support domains on both sides of the line feature, we constructed an initial matching matrix and screened the correct matching pairs based on the geometric properties of the line feature, ensuring the accuracy and robustness of line feature matching.

**Figure 8:**
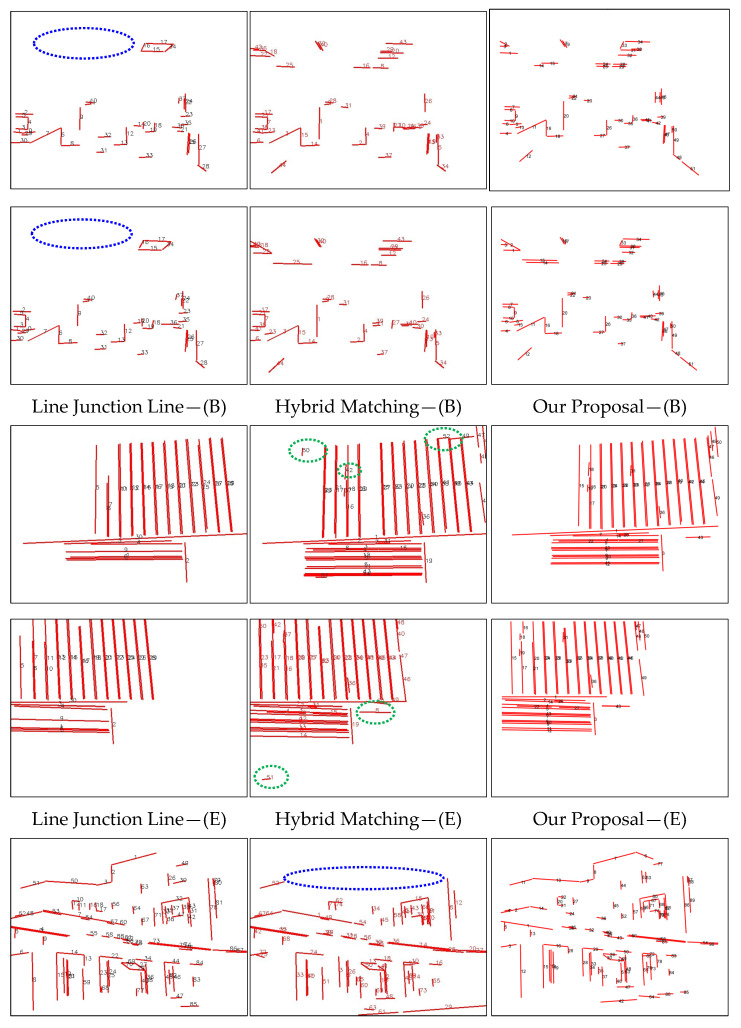

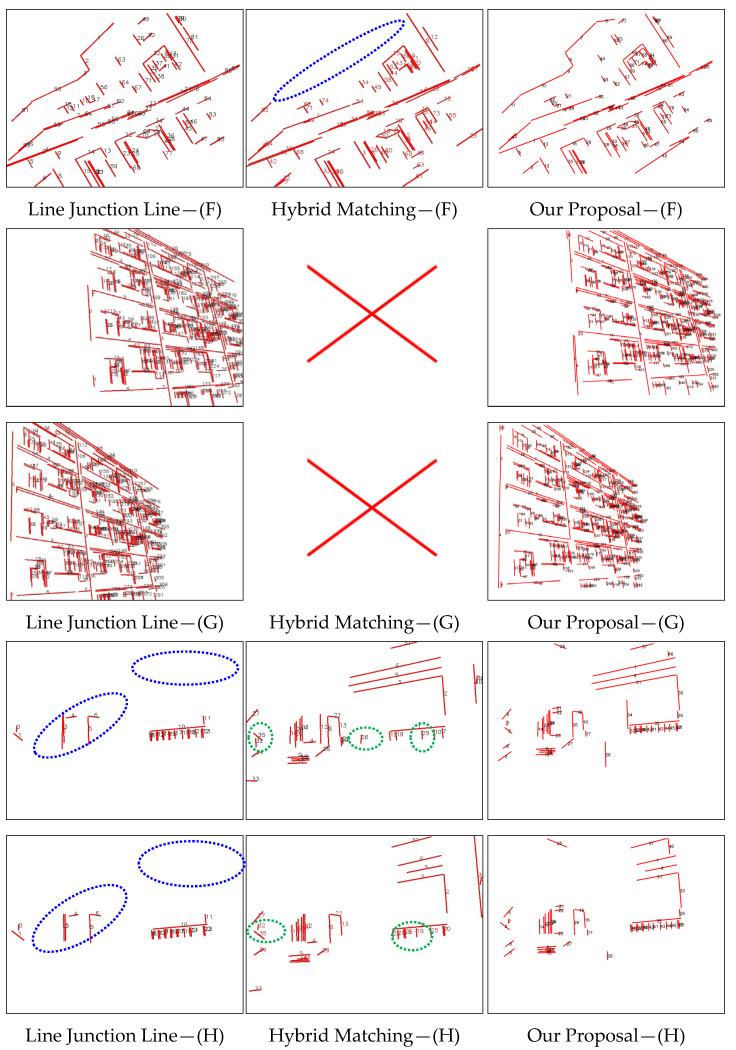
Matching performance comparison across among image pairs (B), (E), (F), (G), and (H).

Figure 8 presents several visualized results from line feature matching algorithms, including Line Junction Line, Hybrid Matching, and our proposed method. The figure illustrates line matching performance under challenging indoor conditions: weak texture, low illumination, rotation, and occlusion. To emphasize differences in the results, we mark obvious mismatches with green dotted ellipses and missing match areas with blue dotted ellipses. According to the visualized results shown in figure above, Line Junction Line or Hybrid Matching suffers significant matching losses in cases of line features in occlusion situations (E), low-texture line features (B), and sparse line detection in dim scenes (H). Meanwhile, Hybrid Matching requires homography matrices to filter line matches. Since these matrices are estimated from line endpoints, their accuracy affects robustness, leading to failure in the image pair (G).

In contrast, our proposal algorithm is not affected by the factors analyzed above. By incorporating robust point features to augment line feature matching and exploiting the geometric constraints of lines more effectively, our approach achieves significantly improved performance compared to the referenced works [23,27].

### 4.3. Comparison Between Our Proposal and Learning-Based Algorithms

We further evaluated our method against state-of-the-art deep learning-based line matching techniques, including LineTR [36], WLD [39], and SOLD2 [35]. LineTR is an end-to-end 3D line feature tracking algorithm based on Transformer, inspired by point feature tracking methods. It combines line feature detection (LSD) [42] with 3D line feature matching in space, leveraging attention mechanisms to enhance cross-view line feature association. WLD uses wavelet transform to extract local multi-scale features of lines and optimizes feature representation through learning methods to improve the discriminative power of descriptors. SOLD2 is a self-supervised line feature detection and description algorithm, designed to robustly extract and match line features in challenging scenarios (e.g., occlusion, illumination variation). For a fair comparison, we conducted experiments using the LSD line detector (the same as LineTR’s backbone) on image pairs (A)~(I). The quantitative results, presented in Table 2, highlight our method’s superior performance (bold indicates optimal results). Entries marked with “-” denote cases with fewer correct matches, while “*” indicates lower matching accuracy.

From the above results, it is evident that LineTR, WLD, and SOLD2 algorithms all achieve a higher number of matched line features, obtaining the optimal performance on four out of the nine datasets. However, their successful matches are predominantly observed in scenarios with minimal rotation and translation between image pairs. In contrast, their robustness declines significantly under challenging conditions such as large viewpoint changes, scale variations, or severe rotations. Although our proposal algorithm’s matching performance is somewhat inferior to these deep learning-based methods, it does not rely on specialized hardware and exhibits stronger generalization capabilities compared to LineTR, WLD, and SOLD2. While the total number of line feature matches in some sequences may be fewer than those achieved by deep learning-based methods, our approach consistently delivers superior matching accuracy.

**Figure 9:**
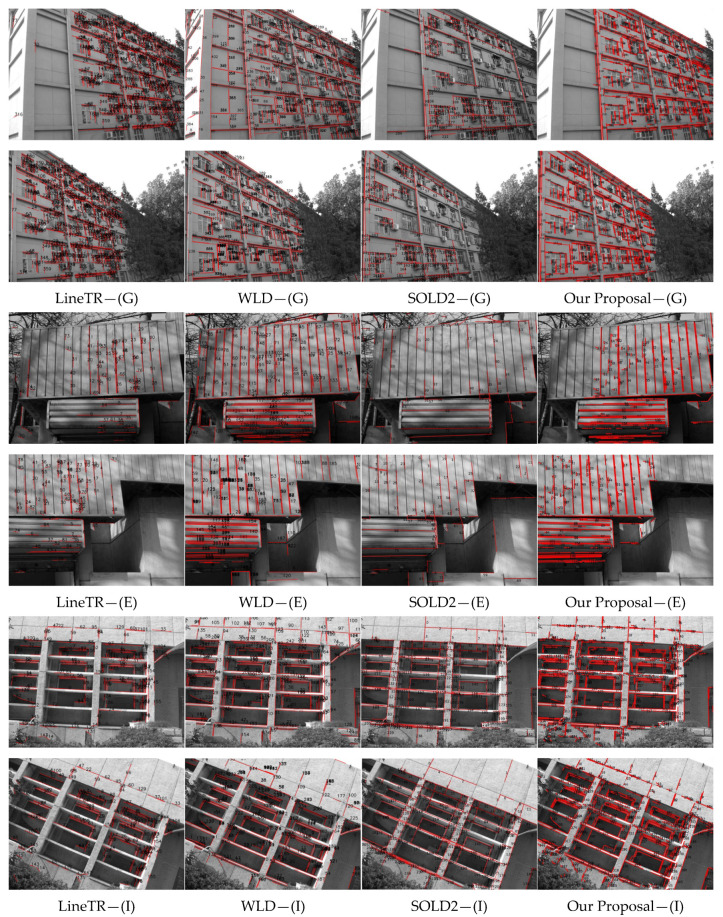
Line feature matching results comparison: Our method versus LineTR, WLD, and SOLD2.

Figure 9 demonstrates the features matching results under several special scenarios. Notably, both algorithms exhibit significantly low matching accuracy on image pair (E). However, our method maintains a robust performance advantage when handling other cases with substantial viewpoint variations and rotations. In image pair (E), specifically, while both LineTR and SOLD2 algorithms generate numerous line feature matches, a significant portion of these matches are erroneous. Although WLD algorithm matches more line features in image pairs (G), (E), and (I), there are actually many mismatches. This phenomenon may be attributed to limitations in the diversity of training samples utilized for these models, highlighting an inherent constraint of learning-based feature matching approaches.

### 4.4. Analysis of Ablation Experiment

To validate the effectiveness of the two-term point–line invariant constructed for the line feature matching algorithm in this paper, we performed ablation experiments and conducted both qualitative and quantitative comparative analysis. In this section, to demonstrate the proposed algorithm, the ablation experiments were conducted on several typical image pairs involving rotation, low texture, and occlusion situation. Specifically, image pairs (B), (E), (F), and (I) from the experimental dataset were selected for testing. The experiments were carried out by incrementally adding each of the two-term point–line invariant, and the results are summarized in Table 3. The comparison is still based on the matching performance (*MP*) as the evaluation metric. In Table 3, this “*Invariant 1*” denotes the angle invariant, “*Invariant 2*” denotes the distance invariant, and “*Invariant 1 + 2*” denotes a combination of the two invariants (e.g., angle + distance).

It is not difficult to find that the number and accuracy of matching line features based on a single invariant may not be optimal. When we combine two invariants to match constrained line features, we can obtain the maximum number of line feature matches, and the matching accuracy of this line feature is also optimal.

**Figure 10:**
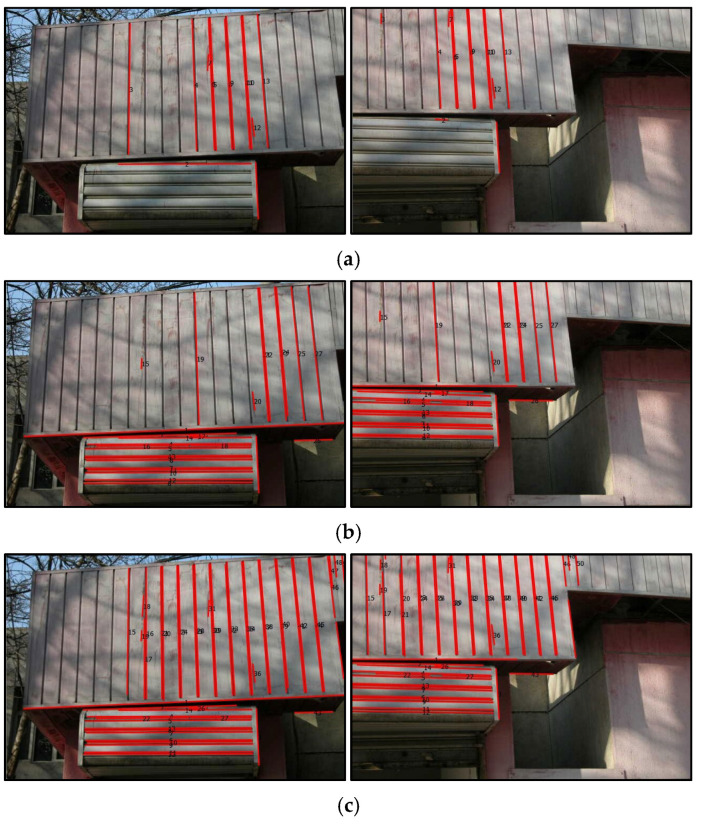
Comparison results of the ablation experiment on the image pair (E): (**a**) The matching results based on angle invariant; (**b**) the matching results based on distance invariant; (**c**) the matching results based on distance and angle invariant.

Figure 10 lists the corresponding visualization results for the invariant-constrained ablation experiments example based on image pair (E). Based on the matching results shown in the figure below, we know that the number of line feature matches is not optimal when only using point–line invariant condition 1 or invariant condition 2. This indicates that there is a problem of overscreening line features based solely on two terms of point–line invariants. After adjusting the weights of two terms of invariant conditions appropriately, the matching quantity and accuracy of line features were significantly improved, which indirectly proves the effectiveness of our algorithm.

### 4.5. Line Feature Matching in Illumination Sensitive or Blurred Scenes

**Figure 11:**
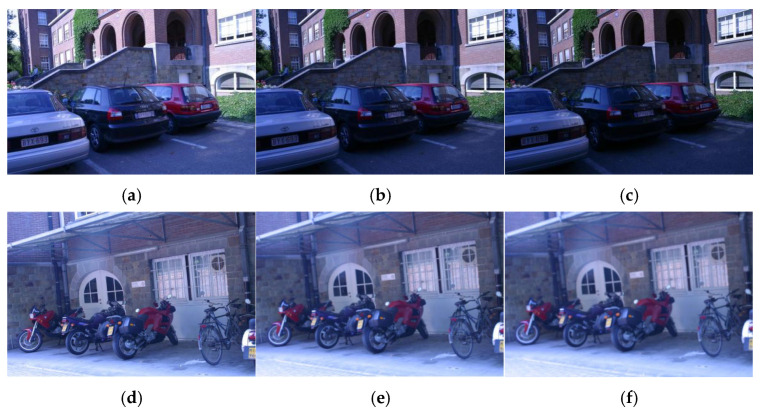
The examples of images under different conditions selected for the experiment. Images (**a**–**c**) demonstrate a gradual reduction in lighting variations, while images (**d**–**f**) exhibit a progressive increase in blurring.

Given that the IILB line feature descriptor proposed in ref. [24] is specifically designed for matching line features in scenes sensitive to light or affected by varying illumination conditions, we conducted additional comparative experiments to evaluate the robustness of our proposed algorithm under such challenging scenarios. Using the image sequence from the previously described image pairs, we compared six pairs of images under different lighting conditions, labeled as (a) to (e) (Figure 11). Specifically, the pairs (a,b), (a,c), (b,c), (d,e), (d,f), and (e,f) were selected to represent significant variations in lighting or blurring conditions, where the first image in each pair was matched against images with significant illumination or clarity differences. The comparative results are summarized in Table 4, with the MP metric employed to assess the matching performance.

As shown in Table 4, the initial matching pairs are obtained through bidirectional matching based on descriptors, followed by a one-by-one calculation of descriptor similarity to derive the final matching pairs. In contrast, ref. [24] applies a length filtering process to the line feature extraction results, which significantly reduces the number of line feature matches compared to the direct use of LSD line feature extraction in this study. From Table 4, it is evident that the proposed algorithm surpasses the IILB descriptor in ref. [24] in two key aspects: the number of feature-matching pairs and the *MP* indicator.

## 5. Discussion Analysis and Application

### 5.1. Discussion and Anaylsis

According to these above qualitative and quantitative experimental results, it is evident that our algorithm can complete the calculations for all image pair sequences, which indirectly demonstrates the robustness and matching accuracy of our algorithm. As shown in Section 4.2 and Section 4.3, our algorithm has a distinct advantage in matching accuracy. Among the nine sets of image pairs, we managed to obtain six optimal results in terms of both the number of matching features and accuracy in several sets of data. Since our algorithm is based on the geometric distance relationship between point and line features, the algorithm depends on the robustness of point feature matching to a certain extent. For example, SOLD2 is an adaptive line feature detection and matching algorithm that can detect and match more lines in most data test pairs. However, deep learning still relies on training samples and hardware facilities, and once the sample diversity is insufficient, the matching results of such algorithms will plummet.

In the experiments focused on line feature matching under the varying illumination and blur conditions, we conducted corresponding experiments across diverse lighting and blurring scenario sequences. Our algorithm achieved optimal results in three out of these tested images pairs. In addition, we also gave a comparison of matching results from image pair (H) in Section 4.2 and Section 4.3. Notably, in dimly lit scenes, our algorithm consistently outperformed others across all 3 experimental sets. However, in sequences with significant blur, point feature mismatching occasionally occurred, leading to inaccuracies in the calculation of the homography matrix and, consequently, a reduction in matching accuracy.

In fact, our algorithm is not optimal in all aspects, our algorithm is based on Matlab language, where there is a large number of matrix loop traversal operations embedded in the algorithm. The time efficiency of our algorithm is related to the pixel size of the image pair and the number of detected line features. For example, the image pairs (C) and (D) shown in Figure 6 consume less than 30 s, while (B) and (E) will not exceed 1 min. Furthermore, the matching of point features must be completed first; the average matching time of the algorithm for image data with a total number of pixels is not very large, but for images with large pixels (such as drone images), the algorithm needs more time. This is where our algorithm needs to improve; we will optimize the code and rewrite it to C or C++ version to improve the speed of the algorithm in future works.

### 5.2. Sparse Reconstructions Based on 3D Lines

**Figure 12:**
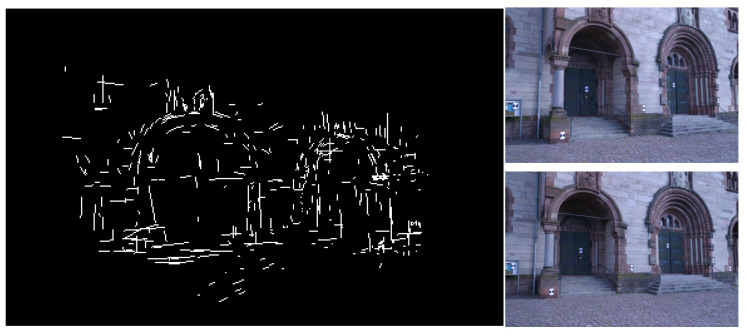
3D sparse line reconstruction results from matched line features.

To make our proposed line feature matching algorithm applicable in practice, we extracted and reconstructed three-dimensional line features based on a high-resolution ground image dataset (Herz-Jesu) [43] and combined our line feature matching results. Figure 12 displays the results of Herz-Jesu. We detected and matched these line features in two views; it requires more data processing time to acquire the 3D line due to the high image resolution of these data. As shown in Figure 12, it can be seen that, based on our algorithm, we basically described the structure information of the scene based on line features, which is useful for 3D reconstruction.

## 6. Conclusions

We proposed an innovative line feature matching algorithm that leverages geometric relationships between feature points and lines to construct the invariant for robust line matching. By introducing the distance metric of line-to-line (i.e., endpoint and midpoint distance) across diverse scenarios, our method establishes strong geometric constraints, effectively eliminating mismatches while significantly enhancing both accuracy and robustness. Extensive experimental evaluations—including qualitative and quantitative analyses—demonstrated that our algorithm outperforms most line feature matching methods. While achieving excellent matching accuracy, there is still a lot of room for improvement in the efficiency of our algorithm due to our use of the MATLAB platform, especially in the iterative calculation of homography matrices and matching matrix processing. Our future work will focus on terms of algorithmic refinement and code-level optimizations to improve efficiency without compromising performance. The potential enhancements include adopting parallel computing techniques and advanced matrix operation optimizations to accelerate processing.

## Figures and Tables

**Table 1:** Quantitative comparison of matching performance between the proposed algorithm and reference methods [23,27].

	Line Junction Line		Hybrid Matching		Our Method	
	(I_1-line_,I_2-line_,)/MS	MC/MP	(I_1-line_,I_2-line_,)/MS	MC/MP	(I_1-line_,I_2-line_,)/MS	MC/MP
(A)	[464,202]/90	87/0.97	—/—	—/—	[464,202]/109	**108/0.99**
(B)	[69,61]/44	44/1.0	[70,63]/47	44/1.0	[70,63]/51	**51/1.0**
(C)	[35,27]/12	12/1.0	[34,34]/21	21/1.0	[34,34]/24	**24/1.0**
(D)	[24,27]/16	16/1.0	[23,24]/17	17/1.0	[23,24]/17	**17/1.0**
(E)	[163,112]/31	31/1.0	[163,112]/52	45/0.86	[163,112]/50	**50/1.0**
(F)	[136,119]/89	89/1.0	[139,113]/75	75/1.0	[139,113]/90	**90/1.0**
(G)	[481,421]/337	**337/1.0**	—/—	—/—	[485,421]/336	336/1.0
(H)	[196,59]/24	24/1.0	[196,59]/36	30/0.83	[196,59]/47	**47/1.0**
(I)	[218,214]/167	**167/1.0**	—/—	—/—	[210,213]/165	165/1.0

**Table 2:** Quantitative comparison of line feature matching performance between LineTR, WLD, SOLD2, and our proposed method.

	LineTR (LSD)	WLD (LSD)	SOLD2	Our Method (LSD)
	MS/MC/MP	MS/MC/MP	MS/MC/MP	MS/MC/MP
(A)	121/101/0.83	201/-/*	116/96/0.83	**109/108/0.99**
(B)	172/170/0.98	113/107/0.95	**281/281/1.0**	210/210/1.0
(C)	54/54/1.0	54/48/0.89	**66/66/1.0**	55/55/1.0
(D)	38/38/1.0	44/30/0.68	**72/72/1.0**	38/38/1.0
(E)	86/60/0.70	208/-/*	80/20/0.20	**128/126/0.98**
(F)	78/73/0.94	216/-/*	324/300/0.93	**331/331/0.99**
(G)	368/364/0.99	413/-/*	212/207/0.98	**377/377/1.0**
(H)	153/151/0.99	251/-/*	**245/245/1.0**	190/190/1.0
(I)	156/144/0.92	253/-/*	246/240/0.98	**397/395/0.99**

**Table 3:** Ablation study validating the components of our proposed line matching algorithm.

	*Invariant 1*: (MC/MS/MP)	*Invariant 2*: (MC/MS/MP)	*Invariant 1 + 2*: (MC/MS/MP)
(B)	49/49/1.0	51/51/1.0	51/51/1.0
(E)	12/13/0.92	27/27/1.0	50/50/1.0
(F)	81/81/1.0	83/83/1.0	90/90/1.0
(I)	158/158/1.0	156/156/1.0	165/165/1.0

**Table 4:** Quantitative performance comparison between our proposed algorithm and the method from ref. [24].

	IILB Descriptor	Line Junction Line	Hybrid Matching	Our Method
	MS/MC/MP	MS/MC/MP	MS/MC/MP	MS/MC/MP
(a,b)	126/123/0.98	247/247/1.0	—/—/—	**256/256/1.0**
(a,c)	93/85/0.91	218/218/1.0	—/—/—	**230/230/1.0**
(b,c)	110/110/1.0	221/221/1.0	93/93/1.0	**240/240/1.0**
(d,e)	101/93/0.92	188/188/1.0	—/—/—	**209/209/1.0**
(d,f)	62/54/0.87	**132/132/1.0**	—/—/—	127/127/1.0
(e,f)	74/67/0.95	108/108/1.0	—/—/—	**112/112/1.0**

## Data Availability

The dataset can be found at https://github.com/zcyHHU/Line-Matching-Dataset (accessed on 6 May 2025), and the datasets all are collected by the camera.
